# Proceedings of the International Gap Junction Conference 2015

**DOI:** 10.1186/s12860-016-0097-5

**Published:** 2016-05-24

**Authors:** Juan C. Sáez

**Affiliations:** Centro Interdisciplinario de Neurociencias de Valparaíso, Universidad de Valparaíso, Valparaíso, Chile; Departamento de Fisiología, Pontificia Universidad Católica de Chile, Santiago, Chile

**Fig. 1:**
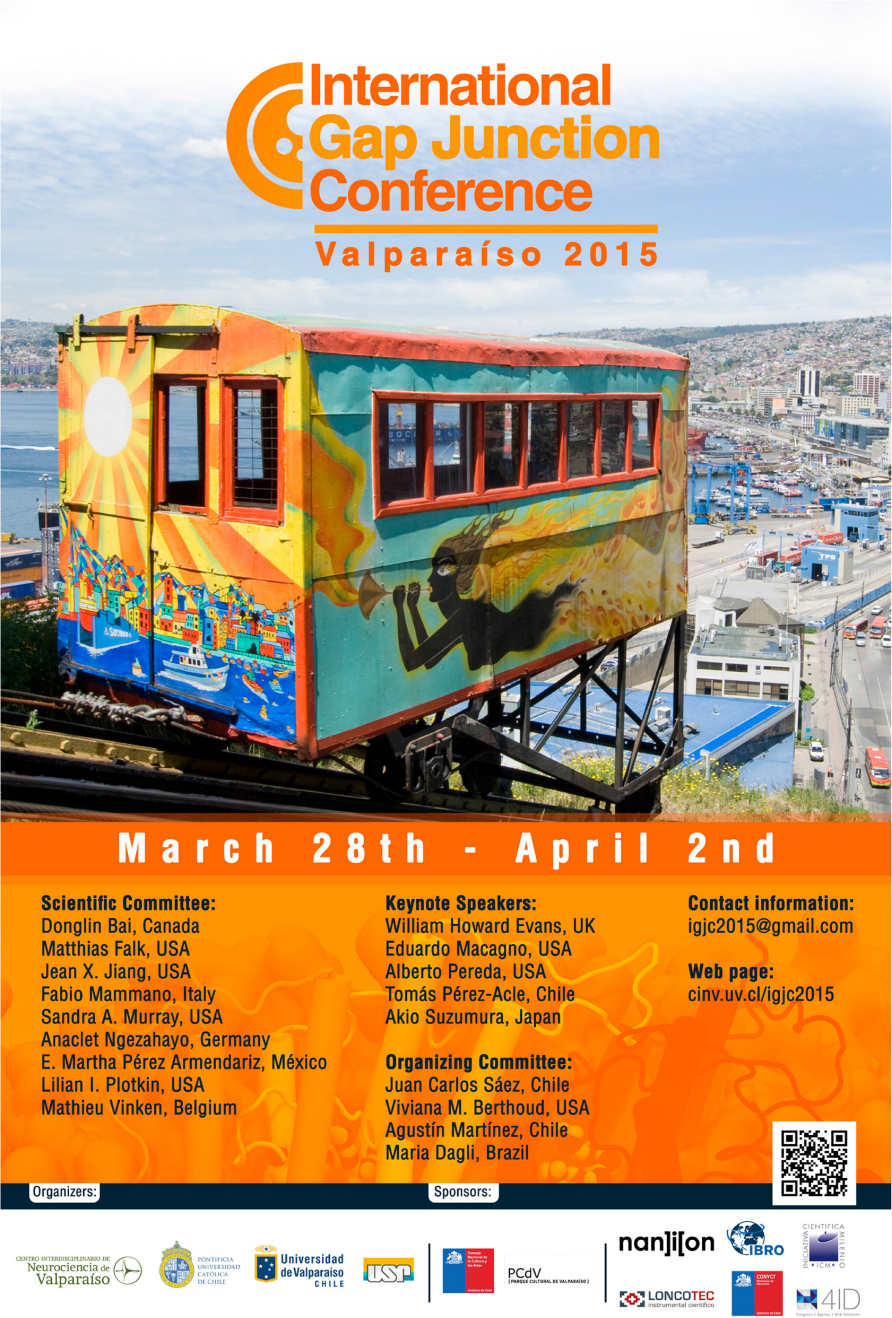
International Gap Junction Conference 2015 poster

From March 28 through April 2, 2015, the scientific community performing research on connexin-, pannexin- and innexin-based channels (gap junction channels and hemichannels) attended the International Gap Junction Conference in Valparaíso, Chile (Fig. 1). The Conference counted with the participation of 147 attendees from 19 different countries. Several topics were presented and discussed in platform and poster sessions, including novel insights into the structure, cell biology, regulation and pharmacology of the channels as well as different mechanisms by which these channels contribute to diverse pathologies. The keynote speakers covered specific topics. Dr. Eric Beyer gave a recount of the changes that took place in the field after the cloning of the first connexin cDNAs. Dr. Tomás Pérez-Acle presented novel findings into the structure-function relationships of different connexin channels. Dr. Eduardo Macagno presented a historical perspective of innexins and emphasized how the identity of the subset of innexins expressed in a neuron determines its morphology and connectivity during development. Dr. Alberto Pereda talked about the structural complexity and dynamic regulation of electrical synapses. Dr. Akio Suzumura presented the latest progress of his group on the use of connexin hemichannel blockers to prevent neuronal degeneration in several neurodegenerative diseases. The participation of undergraduate and graduate students as well as post-doctoral fellows played a significant role in the success of the meeting. Several of them were recognized for their scientific accomplishments and received honorable mentions and prizes. One of the highlights of the Conference was the development of specific modulators of channel and hemichannel activities. These modulators may be useful to further understand the role these proteins play in different physiological and pathological conditions. Some of them have been successfully tested for the treatment of certain pathological conditions in mice and have the potential of being used for the treatment of different human diseases in the future. The main topics discussed at this International Conference have been reviewed by specialists in the field and will be published in two special issues in BMC Cell Biology.

The organizers would like to give special recognition to Drs. Michael Bennett from USA and Klaus Willecke from Germany for their major contributions to the field, and their critical questions and opinions. The scientific community also thanks Dr. Klaus Willecke for making all the mice with targeted deletion or replacement of connexins available to the community. We also acknowledge Mr. Juan Carlos García and his team from the Centro Interdisciplinario de Neurociencias de Valparaíso (CINV) for their invaluable support and assistance with the logistics of the meeting. In addition, we are indebted to the CINV, Pontificia Universidad Católica, GrupoBios, Nanion Technology and Loncotec for their financial support, which covered some of the activities associated with the Conference. Finally, we are thankful to the Chilean Millennium Initiative of the Chilean Government for the CINV (P09-022-F) grant directed by Dr. Ramón Latorre through which we financed several activities and allowed us granting of 43 international student fellowships.

